# Modulation of the Gut Microbiota Structure with Probiotics and Isoflavone Alleviates Metabolic Disorder in Ovariectomized Mice

**DOI:** 10.3390/nu13061793

**Published:** 2021-05-25

**Authors:** Qian Chen, Botao Wang, Shunhe Wang, Xin Qian, Xiu Li, Jianxin Zhao, Hao Zhang, Wei Chen, Gang Wang

**Affiliations:** 1 State Key Laboratory of Food Science and Technology, Jiangnan University, Wuxi 214122, China; 18262282299@163.com (Q.C.); jnwangbotao@foxmail.com (B.W.); wangshunhe@jiangnan.edu.cn (S.W.); qian_x@hotmail.com (X.Q.); lixiu@jiangnan.edu.cn (X.L.); zhaojianxin@jiangnan.edu.cn (J.Z.); zhanghao@jiangnan.edu.cn (H.Z.); chenwei66@jiangnan.edu.cn (W.C.); 2 School of Food Science and Technology, Jiangnan University, Wuxi 214122, China; 3 International Joint Research Laboratory for Probiotics, Jiangnan University, Wuxi 214122, China; 4 (Yangzhou) Institute of Food Biotechnology, Jiangnan University, Yangzhou 225004, China; 5 National Engineering Research Center for Functional Food, Jiangnan University, Wuxi 214122, China; 6 Wuxi Translational Medicine Research Center and Jiangsu Translational Medicine Research Institute Wuxi Branch, Wuxi 214122, China

**Keywords:** menopause, obesity, estrogen, short-chain fatty acids, microbiota, probiotics, isoflavones

## Abstract

The decrease in ovarian hormone secretion that occurs during menopause results in an increase in body weight and adipose tissue mass. Probiotics and soy isoflavones (SIFs) could affect the gut microbiota and exert anti-obesity effects. The objective of this study was to investigate the effects of probiotics and a diet containing SIF (SIF diet) on ovariectomized mice with menopausal obesity, including the gut microbiome. The results demonstrate that *Bifidobacterium longum* 15M1 can reverse menopausal obesity, whilst the combination of *Lactobacillus plantarum* 30M5 and a SIF diet was more effective in alleviating menopausal lipid metabolism disorder than either components alone. Probiotics and SIFs play different anti-obesity roles in menopausal mice. Furthermore, 30M5 alters the metabolites of the gut microbiota that increase the circulating estrogen level, upregulates the expression of estrogen receptor α in abdominal adipose tissue and improves the production of short-chain fatty acids (SCFAs). A SIF diet can significantly alter the structure of the fecal bacterial community and enrich the pathways related to SCFAs production. Moreover, 30M5 and a SIF diet acted synergistically to effectively resolve abnormal serum lipid levels in ovariectomized mice, and these effects appear to be associated with regulation of the diversity and structure of the intestinal microbiota to enhance SCFAs production and promote estrogen circulation.

## 1. Introduction

As ovarian function declines, estrogen production and estrogen receptor function also decline, leading to abdominal fat accumulation and metabolic abnormalities in menopausal women [1]. Estrogen plays an important role in controlling food intake and body weight [2]. Hormone replacement therapy can reverse the abnormal lipid metabolism induced by estrogen deficiency [3], but its effectiveness is limited by many factors, including dosage, age, duration and symptoms [4]. Moreover, numerous investigations have found that hormone replacement therapy can lead to increased risks of breast cancer and ovarian cancer [5,6].

The gut microbiome, which consists of the microbes in the intestinal tract and their metabolites, plays an important role in controlling the host’s health status [7]. The intestinal community develops throughout the host’s lifespan by establishing mutual symbiotic relationships that favor co-existence [8]. The gut microbiome can influence the host by secreting metabolites into the bloodstream [9], and alterations of the composition of the gut microbiota can significantly affect the development of chronic disorders [10]. It has been reported that alterations in the gut microbiota induced by estrogen deficiency are closely related to obesity and metabolic disorders in menopausal women [9]. The gut microbiome has been proposed as a possible therapeutic target to address metabolic disorders associated with menopause [9].

Probiotics and soybean isoflavones (SIFs) have the potential to regulate the gut microbiota. Probiotics are live bacteria that can benefit the host by modulating the gut microbiota and immunogenic responses and by producing beneficial substances [11]. Probiotic bacteria have been used strategically to target the gut microbiota and thus combat metabiotic disorders [12]. In particular, strains of *Bifidobacterium* and *Lactobacillus* showed outstanding performance in alleviating obesity and insulin resistance [13,14]. Phytoestrogens have a chemical structure similar to that of mammalian estrogens and exert estrogen-like effects [15]. As one of classes of phytoestrogens, SIFs also have an estrogenic effect. When metabolized by the gut microbiota, SIFs can exert full estrogenic activity and play a beneficial role in the reduction of menopausal obesity [16,17,18,19].

In this study, a mouse model of menopause was used to investigate the synergistic effects and potential mechanism of action of probiotics and SIFs in the treatment of menopausal obesity. Obesity-related changes in lipid metabolism and in the intestinal microbiome are highlighted in this study.

## 2. Materials and Methods

### 2.1. Preparation of Probiotic Strains

*Lactobacillus plantarum* 30M5 was isolated from fermented food and *Bifidobacterium longum* 15M1 was isolated from a healthy woman’s feces. No human experiment was involved in this study and written informed consent was obtained from the participant. The collection of fecal samples had no risk of predictable harm or discomfort to the participant. Both strains were stored at the Culture Collection of Food Microorganisms in Jiangnan University (Wuxi, Jiangsu province, China). Moreover, 30M5 was cultured in de Man, Rogosa and Sharpe (MRS) broth at 37 °C under aerobiotic condition and 15M1 was cultured in MRS broth containing 0.05% (*w/w*) L-cysteine at 37 °C under anaerobic condition. For the actual animal experiment, strains were collected and re-suspended in phosphate buffer saline (PBS) and prepared weekly to ensure the number of living bacteria.

### 2.2. Animal and Ovariectomy

**Figure 1:**
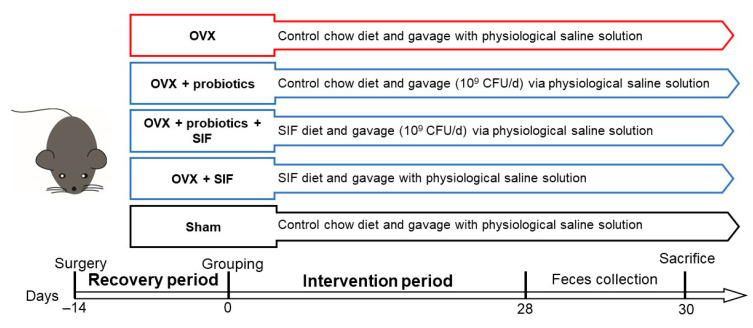
Animal trial design. OVX: Ovariectomy; Sham: Sham-operation.

Animal experimental procedures were performed in accordance with the Experimental Animal Management and Animal Welfare Ethics Committee of Jiangnan University (Protocol number: JN. No 20200430c0720730(002)). Specific-pathogen free (SPF) level C57BL/6N mice (female, 8 weeks old, 18–20 g, *n* = 42) were obtained from Beijing Vital River Laboratory Animal Technology Co., Ltd., Beijing, China. All mice were housed in pathogen-free plastic cages and maintained on a controlled 12 h light/dark cycle at room temperature of 24 °C with free access to food and water. For the first week mice were acclimatized, then all mice either underwent bilateral ovariectomizing to produce an estrogen-deficient state or a sham surgery. After the recovery period of two weeks, ovariectomized mice were randomly divided into six groups according to diet and treatment for one month (six mice per group). Detailed grouping information of the animal experiments are shown in Figure 1. Table 1 presents the composition of each diet. Body weight and food intake were measured weekly after grouping. One month later, all mice were fasted and sacrificed. After sacrificing, the abdominal fat and uterus were dissected and weighed. Additionally, the abdominal fat was immediately frozen and stored at −80 °C until further analysis.

### 2.3. Detection of Sex Hormones Levels in Serum

Mice were deeply anesthetized with 3% pentobarbital sodium (40 mg/kg) and blood were rapidly collected [21]. The serum was prepared by centrifugation at 4000× *g* for 20 min. Serum hormone estradiol (E2), follicle-stimulating hormone (FSH) and luteinizing hormone (LH) levels was measured by enzyme linked immunosorbent assay (ELISA) assay kit (R&D, Elabscience Biotechnology Co., Ltd., Wuhan, China) according to the manufacturer’s instructions.

### 2.4. Determinations of Biochemical Assays

The serum concentrations of total cholesterol (TC), triglycerides (TG), low-density lipoprotein cholesterol (LDL-C), high-density lipoprotein cholesterol (HDL-C) and glucose (Glu) were determined by an automatic biochemistry meter (SELECRTA-E, Vital Scientific). Serum leptin was detected with an enzyme-linked immunosorbent assay kit (R&D, Elabscience Biotechnology Co., Ltd., Wuhan, China). The level of interleukin-6 (IL-6) in abdominal fat was assayed with kits from R&D (Elabscience Biotechnology Co., Ltd., Wuhan, China).

### 2.5. RT-qPCR for Estrogen Receptor (ER) α in Abdominal Adipose Tissue

RNA was extracted from homogenized adipose tissue samples with an ultrapure RNA Kit (CoWin Bioscience, China). Then HiScript III RT SuperMix for real-time quantitative reverse transcriptase polymerase chain reaction (qPCR) and ChamQ Universal SYBR qPCR Master Mix from Vazyme Biotech Co., Ltd., (Nanjing, China) were used to generate the cDNA and perform quantitative PCR. The gene specific forward and reverse primers were synthesized by Sangon Biotech Co.,Ltd (Shanghai, China). Gapdh was used to normalize relative amounts of mRNA. Primer sequences used in this study were as follows: ERα: forward 5′- CCGTCTTACTGTCTCAGCCC-3′, reverse 5′-CGAGTTACAGACTGGCTCCC-3′; Gapdh: forward 5′-AGGTCGGTGTGAACGGATTTG-3′, reverse 5′-TGTAGACCATGTAGTTGAGGTCA-3′. The 2-^ΔΔCt^ value was calculated to quantify the relative expression mRNA level.

### 2.6. Feces DNA Isolation and 16S rRNA Gene Sequence Analysis

Feces were collected before sacrificing and stored at −80 °C. As described previously [22], microbiota genome DNA in feces was extracted by FastDNA Spin Kit for Feces (Cat No. 16570200, MP Bio, Solon, OH, USA). All operational procedures were carried out according to the instructions. Universal primers 515F and 806R were then used to amplify genes via polymerase chain reaction. TIANgel Mini Purification Kit (TIANGEN, Beijing, China) and Qubit dsDNA HS Assay Kit (Life Technologies Corporation, Carlsbad, CA, USA) were used to purify and quantify the PCR products. The 16S rRNA gene amplicon sequencing library was prepared according to the protocol of TruSeq DNA LT Sample Preparation Kit (Illumina, Santiago, CA, USA) and sequenced on an Illumina MiSeq PE300 platform (Illumina, San Diego, CA, USA). Raw sequences were imported into QIIME 2 (version 2020.11) for further analysis.

### 2.7. Measurement of Short-Chain Fatty Acids (SCFAs)

Acetate, propionate and butyrate contents in feces were measured using the gas chromatograph-mass spectrometer (GCMS-QP2010 Ultra system, Shimadzu Corporation, Kyoto, Japan) [23]. Before sample treatment, fecal samples were freeze dried and weighed. SCFAs in samples were extracted by diethyl ether.

### 2.8. Statistical Analysis

Statistical analysis was performed using GraphPad Prism (version 6.01). The results were presented as the means ± standard deviations (SD). Significant difference between study groups were evaluated using ordinary one-way analysis of variance (ANOVA) and Uncorrected Fisher’s LSD multiple comparison test. The mean of each group was compared with the mean of the ovariectomized (OVX) group. Criterion for significance used was a threshold of *p* < 0.05 in all comparisons. Statistical analysis of metagenomic profiles (STAMP, version 2.1.3) and SIMCA-P (version 14.0.1) were used for the metagenomics data analysis. Principal co-ordinates analysis (PCoA) and linear discriminant analysis effect size (LEfSe) were conducted using R (version 3.6.2). Using PICRUSt2, the functional genes were predicted by the Kyoto Encyclopaedia of Genes and Genomes (KEGG) database annotated gene functions.

## 3. Results

### 3.1. Administration of Probiotics and a SIF Diet Alleviated Metabolic Dysregulation and Inflammation Induced by OVX

**Figure 2:**
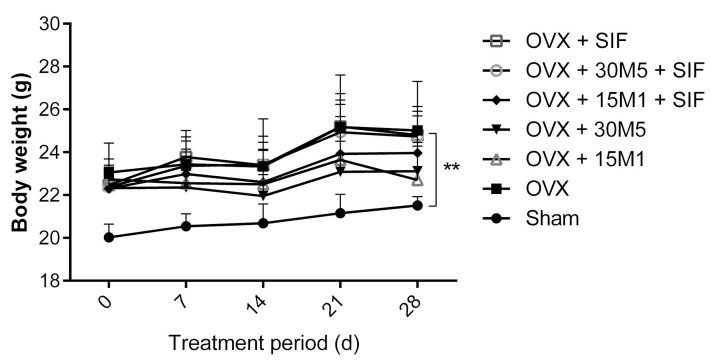
Evolution of body weight during treatment. Data present as the means ± standard deviations (SD).

During the experiment, the mice in both the sham-operated (sham) and ovariectomized groups exhibited increases in body weight. As shown in Table 2, the sham group exhibited an increase in body weight of 7.480% ± 1.938% during the treatment period, whilst the OVX group exhibited a gain of 10.83% ± 2.025%. Although this between-group difference in weight gain did not seem significant, the mice in the OVX group (22.54 ± 0.9310 g) were heavier than those in the sham group (20.14 ± 0.6968 g) before the intervention (Figure 2). Because all mice weighed between 18 and 20 g before surgery and had been randomly assigned to the operation groups (sham or OVX) according to body weight, the findings indicate that mice in the ovariectomized groups gained weight more rapidly than those in the sham group, especially in the period after surgery. However, probiotic treatment alone slowed the rate of weight gain among ovariectomized mice (Figure 2). The body weight of mice in ovariectomized groups was significantly bigger than those in the sham group (*p* < 0.01). Table 2 shows that the weight gains of mice in the OVX + 15M1 (0.420% ± 3.602%), OVX + 30M5 (5.666% ± 1.802%) and OVX + 15M1 + SIF groups (5.654% ± 2.678%) were significantly less than those in the OVX group (10.83% ± 2.025%). Interestingly, a SIF diet seemed to weaken the ability of probiotics in slowing down the body weight gain, the mice in the OVX + 15M1 + SIF group (5.654% ± 2.678%) showed a higher body weight gain than those in the OVX + 15M1 group (0.420% ± 3.602%). Furthermore, the gain in body weight in the OVX + 15M1 group appeared to be unrelated to food consumption, as mice in this group exhibited the least weight gain but a strong appetite. On the other hand, a significant accumulation of abdominal adiposity was seen in ovariectomized mice as well.

**Figure 3:**
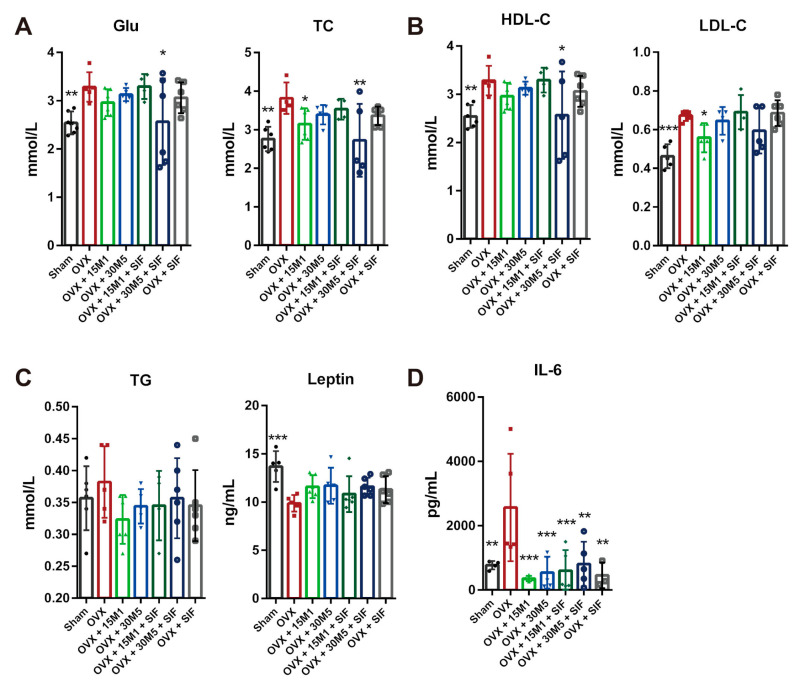
Effect of probiotics and a SIF diet on lipid metabolism and inflammation. (**A**) Glucose (Glu) and Total cholesterol (TC) levels in serum. (**B**) High-density lipoprotein cholesterol (HDL-C) and Low-density lipoprotein cholesterol (LDL-C) levels in serum. (**C**) Triglycerides (TG) and leptin in serum. (**D**) IL-6 level in abdominal fat. * *p* < 0.05, ** *p* < 0.01, *** *p* < 0.001 compared with the OVX group. Data present as the means ± SD. Significant difference between study groups were evaluated using ordinary one-way analysis of variance (ANOVA) and uncorrected Fisher’s LSD multiple comparison test.

Figure 3 lists the parameters related to metabolic function as measured in the serum. Increases in Glu, LDL-C, HDL-C, TG and TC, and a decrease in leptin were exhibited in the OVX group relative to the sham group. Mice in the OVX + 30M5 + SIF group had significantly reduced serum concentrations of Glu, TC and HDL-C (Figure 3A,B), while those in the OVX + 15M1 group exhibited significant decreases in the serum concentrations of TC and LDL-C (Figure 3A,B). The serum TG concentrations of mice subjected to OVX were slightly decreased by intervention with probiotics or a SIF diet (Figure 3C). The serum leptin concentrations tended to increase after supplementation with probiotics or a SIF diet, although the trend was not significant (Figure 3C). The IL-6 concentrations were also analyzed to investigate inflammation in abdominal adipose tissue. The IL-6 levels in adipose tissue were significantly increased after OVX, but supplementation with probiotics and SIFs significantly reduced the concentration of this inflammation biomarker (Figure 3D).

### 3.2. L. plantarum 30M5 Elevated Serum Estradiol Concentrations and Promoted ERα Expression in Abdominal Adipose Tissue in Ovariectomized Mice

**Figure 4:**
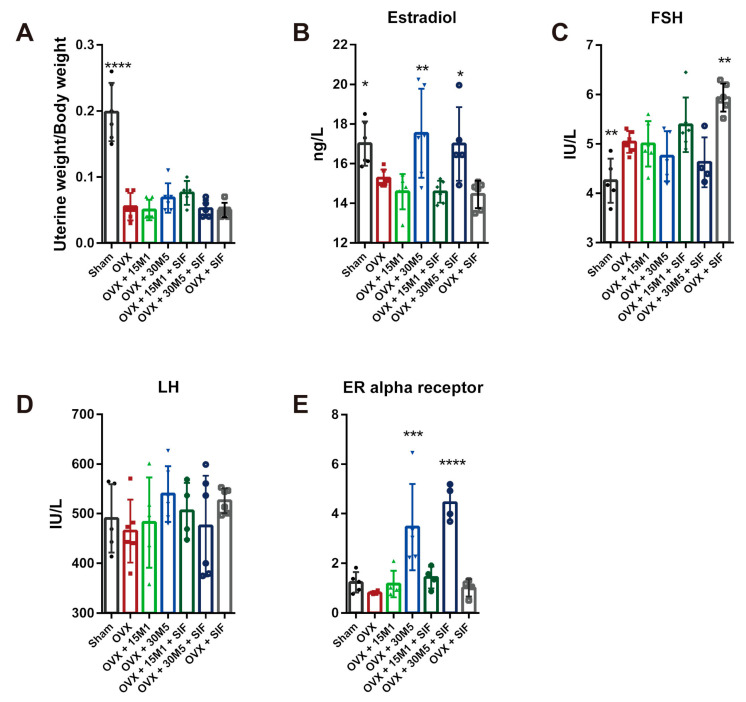
*L. plantarum* 30M5 changes sex hormone levels and up-regulates estrogen receptors. (**A**) Severe atrophy of the uterus in OVX mice. (**B**) Estradiol in serum significantly increased by *L. plantarum* 30M5. (**C**) Follicle-stimulating hormone (FSH) in serum significantly decreased by ovariectomy. (**D**) The level of serum luteinizing hormone (LH). (**E**) The expression of Estrogen Receptor (ER) α in abdominal adipose tissue up-regulated by *L. plantarum* 30M5. * *p* < 0.05, ** *p* < 0.01, *** *p* < 0.001 compared with the OVX group. Data present as the means ± SD. Significant difference between study groups were evaluated using ordinary one-way analysis of variance (ANOVA) and uncorrected Fisher’s LSD multiple comparison test.

A significant decrease in uterine weight indicated the success of OVX (Figure 4A). The serum E2, FSH and LH concentrations were also measured. Figure 4B shows that the serum E2 levels were significantly lower in the OVX group than in the sham group, whereas the E2 concentrations were significantly increased in both the OVX + 30M5 and OVX + 30M5 + SIF groups. A conspicuously lower FSH concentration was found in the sham group than in the OVX group (*p* < 0.01), and a significant increase in the FSH concentration was observed in the OVX + SIF group (Figure 4C). However, as shown in Figure 4C, the FSH concentrations in the OVX + 30M5 and OVX + 30M5 + SIF groups were slightly lower than that in the OVX group, indicating a slight declining trend in the FSH level after the administration of *L. plantarum* 30M5. The serum LH concentration was unaffected by OVX, and no statistical differences in this parameter were seen between the groups (Figure 4D). The increase in estradiol secretion after supplementation with 30M5 prompted us to investigate changes in estrogen receptor expression in the abdominal adipose tissue of mice in the OVX group. The results of qPCR showed that treatment with 30M5 led to a significant up-regulation in the expression of the gene encoding ERα in the abdominal adipose tissue of OVX-treated mice (Figure 4E).

### 3.3. L. plantarum 30M5 and a SIF Diet Exerted Strong Influences on the Intestinal Microbiota, and Altered Microbiota Was Associated with Estradiol Production

**Figure 5:**
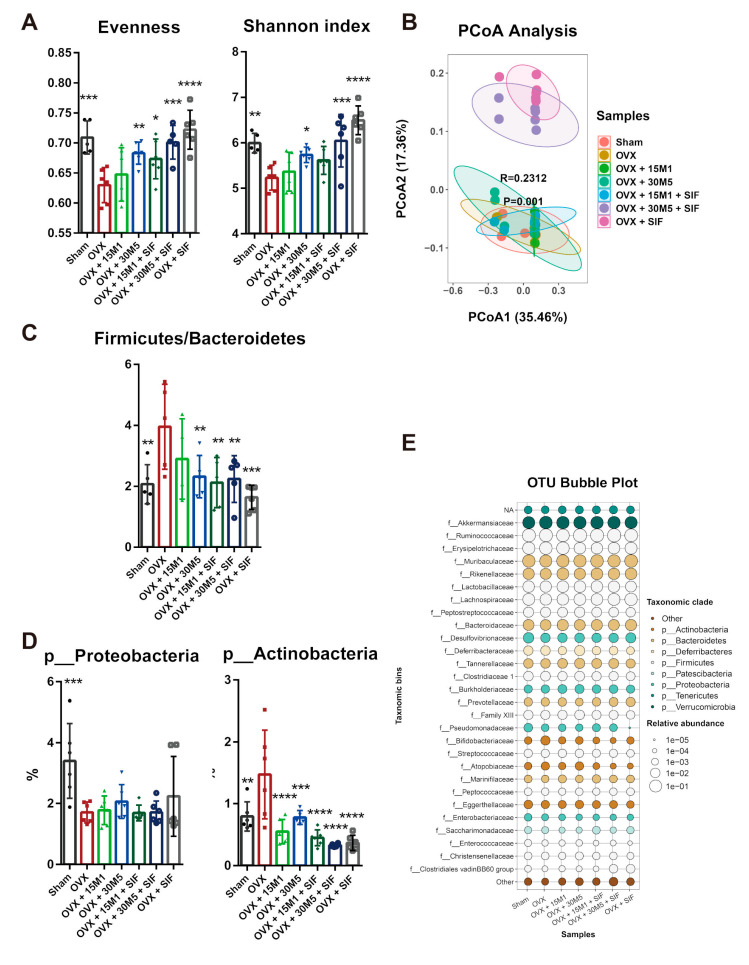
Diversity of intestinal microbiota reduced in ovariectomy and changed by a SIF diet treatment. (**A**) Alpha diversity in fecal samples. (**B**) Beta diversity in fecal samples was measured using principal coordinate analysis (PCoA) of unweighted UniFrac. (**C**)The radio of Firmicutes and Bacteroidetes. (**D**) The changed abundance at phylum level. (**E**) Differential abundance at family level between groups was shown with an OTU bubble plot. * *p* < 0.05, ** *p* < 0.01, *** *p* < 0.001 compared with the OVX group. Data present as the means ± SD. Significant difference between study groups were evaluated using ordinary one-way analysis of variance (ANOVA) and uncorrected Fisher’s LSD multiple comparison test.

To estimate the changes in species diversity after OVX, the bacterial evenness and Shannon index of microbial diversity were calculated for each group of mice (Figure 5A). Ovariectomy significantly reduced evenness, while the decrease in bacterial evenness was partially prevented by the administration of 30M5 and treatment with a SIF diet. The Shannon index was significantly decreased in the OVX group, whereas significant relative increases were observed in the OVX + 30M5, OVX + 30M5 + SIF and OVX + SIF groups. Beta diversity was estimated using weighted and unweighted UniFrac analyses visualized via a principal co-ordinate analysis (PCoA). However, a PCoA plot of only the unweighted UniFrac distance showed the distinct separation of samples (Figure 5B) into two distinct clusters. Samples from the OVX + 30M5 + SIF and OVX + SIF groups differed distinctly from samples from the OVX, sham, OVX + 15M1 and OVX + 30M5 groups, which indicated that the SIF diet had a significant effect on the structure of the intestinal microbiota. It is particularly notable that the OVX and sham groups did not differ in terms of cluster formation exhibited by PCoA. Taken together, these observations suggest that 30M5 supplementation and a SIF diet affect the gut microbiota and introduce greater bacterial diversity after OVX.

The bacterial distribution at the phylum level was also analyzed. No significant difference between groups existed in terms of the preponderant phylum (i.e., Firmicutes or Bacteroidetes). However, the samples from the OVX group had the highest ratio of Firmicutes to Bacteroidetes (Figure 5C). The mice in the OVX group had an obviously lower abundance of Proteobacteria (Figure 5D) and higher abundance of Actinobacteria (Figure 5D) than the sham-operated mice.

Taxonomic bins by family are shown as an OTU bubble plot (Figure 5E). The abundances of Erysipelotrichaceae and Atopobiaceae were increased, whilst those of Ruminococcaceae, Lactobacillaceae, Marinifilaceae, Eggerthellaceae, Enterobacteriaceae and Enterococcaceae were decreased after OVX. The family Bifidobacteriaceae was most abundant in the OVX group. Furthermore, the abundances of Lachnospiraceae, Clostridiales vadinBB60 group and Desulfovibrionaceae were reduced after OVX, whilst a SIF diet further reduced the abundances of these families in the OVX + SIF + 30M5 and OVX + SIF groups.

**Figure 6:**
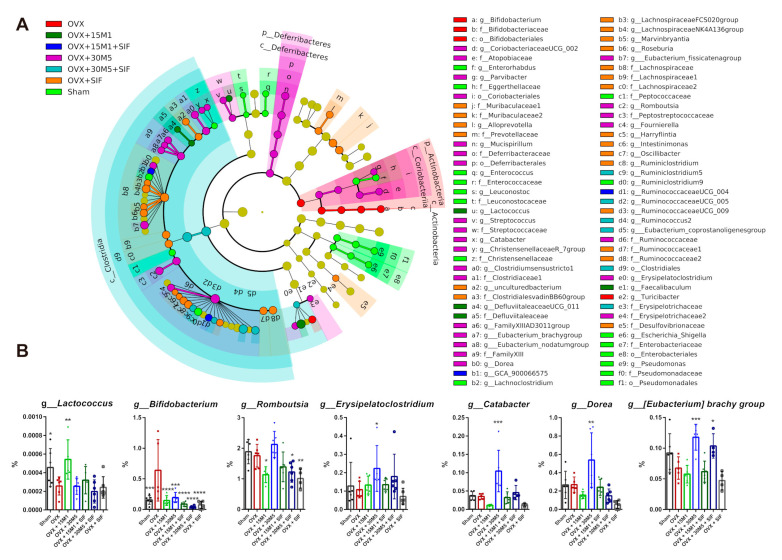
Differential microbial analysis at genus level. (**A**) Phylogenetic tree depicting bacterial taxonomic hierarchy. The tax with log Linear Discriminant Analysis (LDA) scores above 3.00 and *p* score below 0.05 in Wilcoxon test and Kruskal–Wallis test. (**B**) Relative abundance of *Lactococcus*, *Bifidobacterium*, *Romboutsia*, *[Eubacterium] brachy* group, *Catabacter*, *Dorea* and *Erysipelatoclostridium*. * *p* < 0.05, ** *p* < 0.01, *** *p* < 0.001 compared with the OVX group. Data present as the means ± SD. Significant difference between study groups were evaluated using ordinary one-way analysis of variance (ANOVA) and uncorrected Fisher’s LSD multiple comparison test.

At the genus level, differentially abundant taxa were grouped and depicted as a phylogenetic tree using the LEfSe method [24]. Taxa that received log LDA scores above 3.00 and *p* scores below 0.05 in Wilcoxon tests and Kruskal–Wallis tests are shown in Figure 6A. Consistent with the results of the OTU bubble plot, *Bifidobacterium* was the prominent genus in the OVX group. *Enterorhabdus*, *Enterococcus, Leuconostoc*, *Lachnoclostridium*, *Ruminiclostridium 9*, *Escherichia_Shigella* and *Pseudomonas* were more abundant in the sham group than in the OVX groups. *Lactococcus*, Defluvitaleaceae *UCG_011* and *Faecalibaculum* were more abundant in the OVX + 15M1 group. Fourteen bacterial genera, especially *Streptococcus, Catabacter, Dorea, Romboutsia, Fournierella* and *Erysipelatoclostridium*, were identified at higher abundances in the OVX + 30M5 group. The Lachnospiraceae *FCS020* group, the Lachnospiraceae *NK4A136* group, *Alloprevotella, Marvinbryantia, Roseburia, Harryflintia, Intestinimonas, Oscillibacter* and *Ruminiclostridium* were more abundant in the OVX + SIF group. The abundances of *Lactococcus* were lowered by ovariectomy and restored by 15M1 treatment. However, the abundance of *Lactococcus* was decreased by a SIF diet supplement both in mice with or without probiotics treatment (Figure 6B). *Erysipelatoclostridium* and the [*Eubacterium*] *brachy* group were most abundant in the OVX + 30M5 group (Figure 6B).

**Figure 7:**
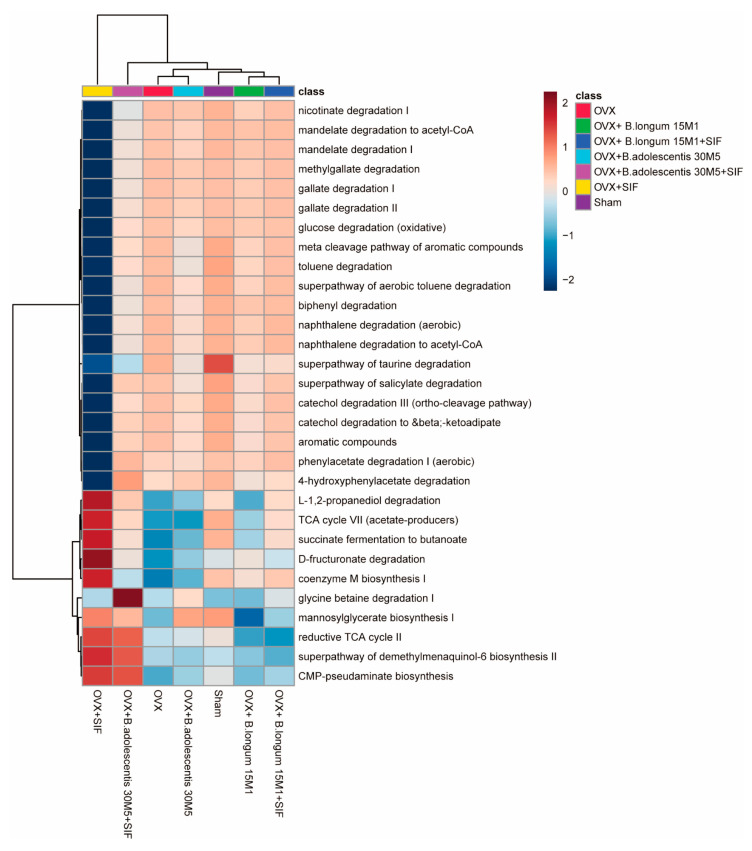
Heatmap of the differential pathways predicted by PICRUSt 2 (phylogenetic investigation of communities by reconstruction of unobserved states).

PICRUSt 2 (phylogenetic investigation of communities by reconstruction of unobserved states) is used to predict the functional composition of a metagenome from marker gene data and a database of reference genomes [25]. In this study, seven differential gene functions were identified between the sham group and the OVX group based on the KEGG (*p* < 0.05, Welch’s test; Figure 7). The following pathways were upregulated in the OVX + SIF, OVX + 15M1 and OVX + 30M5 + SIF groups and down-regulated in the OVX group: L-1,2-propanediol degradation, TCA cycle VII (acetate-producers), succinate fermentation to butanoate, D-fructuronate degradation and coenzyme M biosynthesis I. Interestingly, SIF had a considerable influence on affected pathways, especially in the OVX + SIF group.

**Figure 8:**
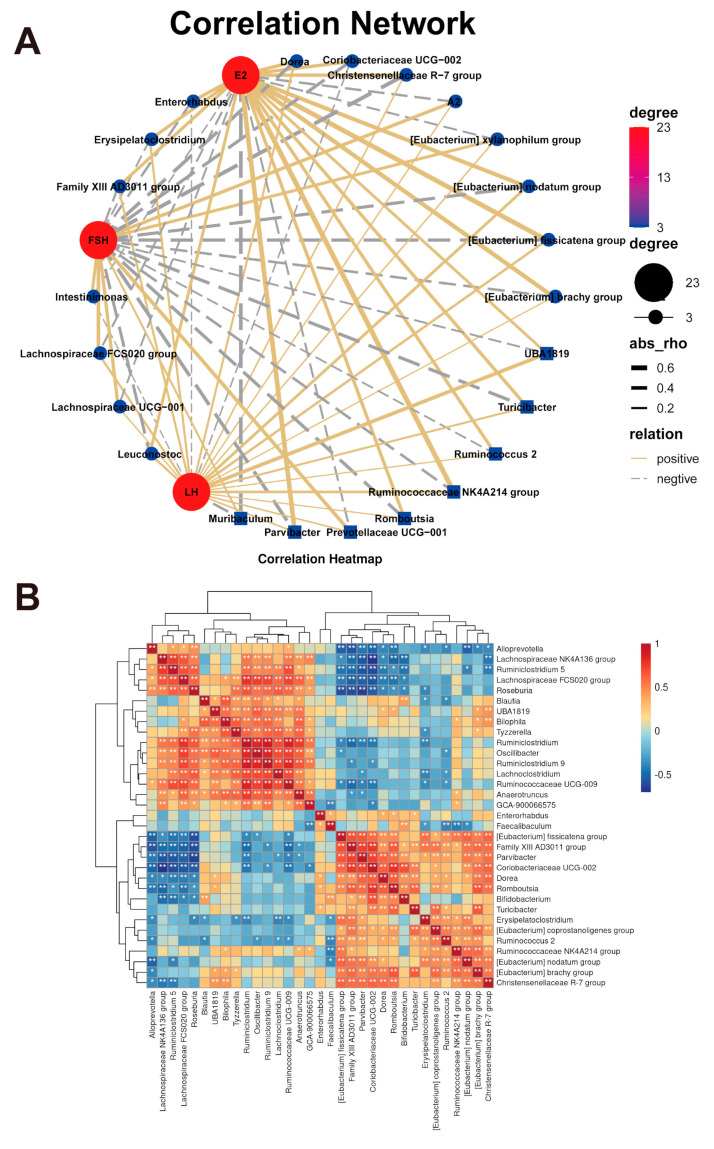
Correlation analysis of microbiota. (**A**) The correlation between sex hormones and intestinal microbiota was shown in network with OmicStudio tools. (**B**) Heatmap of changing microbial relationships.

Correlations between sex hormones (E2, FSH and LH) and the gut microbiota were examined using a correlation network via OmicStudio tools (https://www.omicstudio.cn/tool). As shown in Figure 8A, E2 was shown to be positively correlated with *Dorea*, *Romboutsia*, *Erysipelatoclostridium*, *Enterorhabdus*, *Family XIII AD3011* group, *Ruminococcus 2*, Ruminococcaceae *NK4A214* group, [*Eubacterium*] *nodatum* group, Christensenellaceae *R-7* group and [*Eubacterium*] *brachy* group. Conversely, FSH was shown to be negatively correlated with *Dorea*, *Romboutsia*, *Erysipelatoclostridium* and the [*Eubacterium*] *brachy* group. Gut microbes interact with each other, and correlations between gut microbes were detected. Significant positive linear associations between *Dorea*, *Romboutsia* and the [*Eubacterium*] *brachy* group are shown in Figure 8B. These phenomena indicate that 30M5 treatment influenced the E2-associated gut flora and revealed significant relationships between the altered bacterial genera.

### 3.4. L. plantarum 30M5 Showed a High Production Capacity for SCFAs in OVX Mice

**Figure 9:**
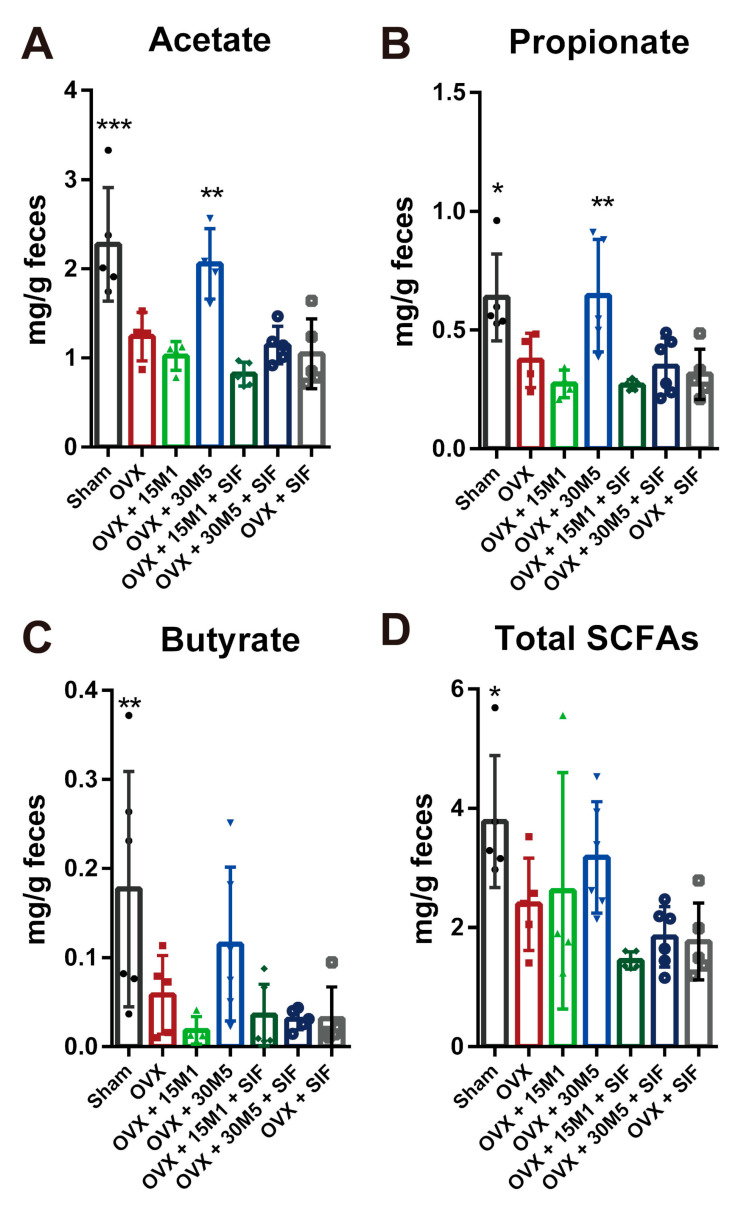
Fecal Short-Chain Fatty Acids (SCFAs). (**A**) Acetate. (**B**) Propionate. (**C**) Butyrate. (**D**) Total SCFAs. * *p* < 0.05, ** *p* < 0.01, *** *p* < 0.001 compared with the OVX group. Data present as the means ± SD. Significant differences between study groups were evaluated using ordinary one-way analysis of variance (ANOVA) and uncorrected Fisher’s LSD multiple comparison test.

Compared with sham group, the levels of acetate, propionate butyrate and total SCFAs in feces of the OVX group were decreased (Figure 9), whilst significant increases in the levels of acetate and propionate were observed in mice in the OVX + 30M5 group compared with the OVX group (Figure 9A,B). The levels of butyrate and total SCFAs tended to increase in mice in the OVX + 30M5 group (Figure 9C,D). Although the SIF diet reduced the levels of SCFAs in the feces of ovariectomized mice, this change was not statistically significant.

## 4. Discussion

Menopause is caused by a lack of estrogen following a decline in ovarian function. Lipid deposition and obesity are common during menopause due to the lack of the protective effects of estrogen that regulate body fat distribution and metabolic health [26]. The composition and metabolic functions of the gut microbiota play important roles in the development of obesity. In this study, probiotics and SIFs were confirmed to exert anti-obesity effects in a model of menopause by targeting the gut microbiome. Furthermore, various strains of probiotics were shown to play different protective roles against menopausal metabiotic disorder.

In this study, conspicuous body weight gain and deposition of abdominal fat occurred after OVX. Supplementation with probiotics and a SIF diet reduced both the serum concentrations of lipids and inflammation in abdominal adipose tissue. Studies have shown that OVX increases the concentrations of TC, LDL-C, HDL-C and Glu [27,28]. In this study, the symptoms of metabolic disorders were significantly mitigated by treatment with 15M1 alone or a combination of 30M5 and a SIF diet. White adipose tissue secretes diverse proinflammatory and anti-inflammatory factors, including adipocytokines such as leptin and the cytokine IL-6 [29,30]. The concentration of circulating leptin was significantly decreased after OVX, while the level of IL-6 in adipose tissue was significantly up-regulated. Leptin, a polypeptide product of a gene associated with obesity, can decrease food intake while increasing energy expenditure [31,32]. Unfortunately, no relationship was observed between food intake and leptin secretion in mice in the OVX group. This finding may indicate that probiotics and a SIF diet did not exert an anti-obesity effect by inhibiting energy intake. However, increasing leptin shown in groups treated with probiotics and a SIF diet showed that probiotics and the SIF diet might be helpful for ovariectomized mice energy consumption promotion. IL-6 is an inflammatory cytokine with adverse metabolic effects [33]. Elevated IL-6 levels have been found in the adipose tissues of both obese mice and obese humans [34,35]. The obvious downward trend caused by treatment with probiotics and a SIF diet in this study suggests that these agents could potentially reduce the inflammatory risk associated with OVX.

The intestinal microbiota plays a vital role in influencing the host via metabolic, nutritional, physiological, immunological and endocrinal functions [36]. In other words, the intestinal microbiota is inseparable from the health of the host. In a similar manner, the distribution of the intestinal microbiota is influenced by various factors, including genes, diseases, the environment, drugs and diet. Several studies have shown that dysbiosis of the gut microbiota occurs in postmenopausal obesity [37,38,39]. The change in sexual hormones is responsible for modulating of the microbiota during menopause [39]. Some changes in the bacterial flora are closely related to the promotion of obesity and adiposity, including an increase in the ratio of Firmicutes to Bacteroides and an increase in the abundances of the Actinobacteria at the phylum level and Erysipelotrichaceae at the class level [40,41,42,43], and these occurred in mice in the OVX group. At the genus level, the advantage of *Bifidobacterium* in the OVX group may be related to the advantage of Actinobacteria in the OVX group because *Bifidobacterium* is classed as Actinobacteria in phylum. PICRUST was used to predict the functional composition of the microbial community [25]. Obvious distinctions in pathways listed in the KEGG were observed between mice in the sham and OVX groups, and most of these changes in the latter group were reversed by a combination of probiotic supplementation and a SIF diet. In particular, sensitive microbial pathways related to SCFA production, including TCA cycle VII (acetate-producers) and succinate fermentation to butanoate, were deficient in the OVX group but enriched by supplementation with probiotics and a SIF diet.

In obese postmenopausal women, probiotics exert a protective effect by modulating the gut microbiota [44,45]. *Lactococcus* in the intestine is a genus of beneficial microbes which negatively associated with obesity [46,47]. Increased abundance of *Lactococcus* in the intestine may be the reason why 15M1 treatment alone was more effective on body weight gain reduction than intervention together with a SIF diet. Supplementation with 30M5 and a SIF diet enriched the abnormal gut microbiota of the mice in the OVX group, as demonstrated by our analyses of bacterial evenness and the Shannon index. Probiotics also affect the ability of the intestinal microbiome to produce metabolites [48]. SCFAs are volatile saturated fatty acids produced in the intestine by the fermentation of carbohydrates and fiber and mainly comprise acetate, propionate and butyrate [49,50]. SCFAs promote the maintenance of intestinal homeostasis and energy metabolism [51]. The fecal SCFAs levels decreased significantly in mice in the OVX group, whilst treatment with 30M5 alone strongly increased the fecal levels of acetate and propionate. The production of estrogen-like metabolites by the gut microbiota is also modulated by probiotics. As an ‘endocrine organ’ of the human body, the gut microbiota interacts with the endocrine system and secretes hormones or hormone-like products to regulate the host’s hormone levels, further influencing the host’s health status [52]. SIFs have chemical structures and functions similar to those of estradiol and can be metabolized by the intestinal bacteria to produce more bioavailable metabolites [53,54]. Probiotics have been reported to promote the bioavailability of SIFs by potentiating the conversion of inactive SIFs to active metabolites [55,56,57]. The synergistic effects of probiotics and SIFs has always been of interest but the lack of conclusions is likely due to the differences of the tested probiotic strains. In this study, the combination of 30M5 and a SIF diet exerted a better protective effect against obesity in the OVX group than either component alone. In other words, 30M5 enhanced the effect of SIFs. However, no synergistic effect was observed with a combination of 15M1 and a SIF diet, which suggests that any synergistic effect of probiotics with SIFs is strain-specific. The most surprising discovery was that probiotic supplementation enhanced the serum estradiol concentration. In addition to the loss of ovarian reproductive function, the uterine weight and serum sex hormone concentrations showed significant reductions after bilateral OVX. Treatment with 30M5 after OVX significantly increased the estradiol concentration and up-regulated ERα expression in abdominal adipose tissue independently of a SIF diet in the mice, which suggests that 30M5 may have the ability to increase circulating estrogen levels. It is well known that the intestinal microbiome is influenced by estrogen; however, the intestinal microbiome also has a significant effect on the circulating estrogen concentration [58]. This influence of the intestinal microbiota is related mainly to the metabolism of estrogens. Conjugated estrogens are excreted into the gastrointestinal lumen within the bile, then de-conjugated by bacterial β-glucuronidase and re-absorbed as free estrogens via enterohepatic recycling [59]. Furthermore, the term ‘estrobolome’ was coined in 2011 as the aggregate of enteric bacterial genes whose products are capable of metabolizing estrogens [60]. We found that 30M5 showed a stronger ability to activate estrogen binding than 15M1 (data not shown). Based on our results, we further hypothesized that 30M5 helped to regulate the intestinal flora and thereby increased the circulating estradiol concentration. Therefore, we analyzed correlations between the intestinal microbiota and estradiol. The order Clostridiales was shown to be directly associated with estrogen metabolism [61]. We found that *Dorea*, *Romboutsia* and (*Eubacterium*) *brachy* group, which all belong to the order Clostridiales, and *Erysipelatoclostridium* increased after treatment with 30M5 and showed significant positive correlations with E2; however, the mechanism by which these bacteria affect estrogen production remains unknown. The results provide new evidence that probiotics can influence the intestinal microbiome to produce estrogen.

## 5. Conclusions

The findings of this study suggest that *B. longum* 15M1 has the potential to alleviate lipid metabolism disorder. Furthermore, a combination of supplementation with *L. plantarum* 30M5 and a SIF diet could attenuate the obesity-related symptoms caused by OVX. Modulation of the imbalance in the intestinal microbiota and increases in the metabolites (SCFAs and estradiol) produced by the intestinal microbiota were shown to be associated with anti-obesity effects in menopausal mice. These findings provide strong proof that the gut microbiome can be used as a therapeutic target to effectively alleviate menopausal metabolic disorders.

## Figures and Tables

**Table 1:** Composition of diet in the animal experiment. Control chow diet: phytoestrogen-purified, soy protein free AIN-93M diet [20]; SIF diet: AIN-93M diet containing 0.1% soy isoflavone (Xi’an Lavia Biotechnology Co., Ltd., Xi’an, China); all diets were prepared by Trophic Animal Feed High-Tech Co., Ltd., Nantong, China.

Ingredient	Group	
	Control Chow Diet (g/kg)	SIF Diet (g/kg)
Cornstarch	427.486	424.986
Casein	200	200
Dextrinized Corn starch	132	132
Sucrose	100	100
Corn oil	40	40
Cellulose	50	50
Mineral mix (AIN-93M)	35	35
Vitamin mix (AIN-93)	10	10
L-Cystine	3	3
Choline bitartrate	2.5	2.5
Tert-butylhydroquinone	0.014	0.014
Isoflavone-rich powder	-	2.5

**Table 2:** Body and food consumption parameters. Body weight gain (%) = (body weight (grouping day 30) − body weight (grouping day 0)) / body weight (grouping day 0). Abdominal adiposity (%) = weight of abdominal adiposity/ final body weight. Data presented as mean ± standard deviations (SD) for variables with normal distribution or median-interquartile range (Q1–Q3) for variables not normally distributed. ** *p* <0.01, **** *p* <0.0001 vs. OVX.

Group	Food Consumption (g/day)	Body Weight Gain (%)	Abdominal Adiposity (%)
Sham	2.709 ± 0.4212	7.480 ± 1.938	2.208 ± 0.3607 **
OVX	2.763 ± 0.2613	10.83 ± 2.025	3.510 ± 0.3814
OVX + 15M1	2.769 ± 0.4632	0.420 ± 3.602 ****	3.223 ± 0.6795
OVX + 30M5	2.493 ± 0.2703	5.666 ± 1.802 **	2.894 ± 0.4978
OVX + 15M1 + SIF	2.642 ± 0.2762	5.654 ± 2.678 **	3.320 ± 0.6107
OVX + 30M5 + SIF	2.833 ± 0.3089	13.660 ± 4.703	3.637 ± 0.9034
OVX + SIF	2.786 ± 0.4192	11.380 ± 1.884	3.563 ± 0.8390
